# Relationships between healthcare staff characteristics and the conduct of vital signs observations at night: Results of a survey and factor analysis

**DOI:** 10.1002/nop2.179

**Published:** 2018-07-16

**Authors:** Alejandra Recio‐Saucedo, Antonello Maruotti, Peter Griffiths, Gary B Smith, Paul Meredith, Greta Westwood, Carole Fogg, Paul Schmidt

**Affiliations:** ^1^ University of Southampton Centre for Innovation and Leadership in Health Sciences Southampton UK; ^2^ Dipartimento di Scienze Economiche Politiche e delle Lingue Moderne – Libera Università Maria Ss Assunta Roma Italy; ^3^ Faculty of Health and Social Sciences University of Bournemouth Bournemouth UK; ^4^ Portsmouth Hospitals NHS Trust Queen Alexandra Hospital TEAMS Centre Portsmouth UK; ^5^ Portsmouth Hospitals NHS Trust Portsmouth UK; ^6^ National Institute of Health Research (NIHR) Collaboration for Leadership in Applied Health Research and Care (CLAHRC) Wessex UK; ^7^ University of Portsmouth – School of Health Sciences and Social Work Portsmouth UK; ^8^ Acute Medicine Unit Portsmouth Hospitals NHS Trust Queen Alexandra Hospital Portsmouth UK

**Keywords:** early warning scores, night‐time surveillance, nurses and midwives, vital signs observations

## Abstract

**Aim:**

To explore the association of healthcare staff with factors relevant to completing observations at night.

**Design:**

Online survey conducted with registered nurses, midwives, healthcare support staff and student nurses who had worked at least one night shift in a National Health Service hospital in England.

**Methods:**

Exploratory factor analysis and mixed effects regression model adjusting for role, number of night shifts worked, experience and shift patterns.

**Results:**

Survey items were summarized into four factors: (a) workload and resources; (b) prioritization; (c) safety culture; (d) responsibility and control. Staff experience and role were associated with conducting surveillance tasks. Nurses with greater experience associated workload and resources with capacity to complete work at night. Responses of student nurses and midwives showed higher propensity to follow the protocol for conducting observations. Respondents working night shifts either exclusively or occasionally perceived that professional knowledge rather than protocol guided care tasks during night shifts.

## 1 INTRODUCTION

The measurement of vital signs is an important component of patient monitoring and is crucial to the prevention of patient deterioration (Le Lagadec & Dwyer, 2017; Smith, 2010). Many hospitals in England use specific monitoring protocols to guide the content and frequency of vital sign measurement and these protocols are based on national guidance (National Institute for Clinical Excellence, 2007). However, studies in the UK, Australia and the USA report accuracy and compliance problems with the completion and documentation of these observations in a timely manner or at all (Tysinger, 2015), especially at night (Griffiths, Recio Saucedo, Schmidt, & Smith, 2014). Issues with surveillance include adherence to monitoring protocols, collection inconsistencies between patient groups, nurses’ views about the importance of patient rest, the negative effects of sleep disruption and consistent implementation of early warning score systems (EWS) (Watson, Skipper, Steury, Walsh, & Levin, 2014).

## 2 BACKGROUND

The underlying reasons for the reduced frequency of measurement of vital signs on general wards at night are unclear and require exploration (Buist & Stevens, 2013). Staffing at night is usually lower than during daytime shifts, with increased patient:staff ratios. Vital signs measurement can be time‐consuming (Mok, Wang, Cooper, Ang, & Liaw, 2015; Yeung, Lapinsky, Granton, Doran, & Cafazzo, 2012), especially when staffing is reduced (Hogan, 2006); challenges are further compounded by alterations in staff skill mix (Wheatley, 2006). These obstacles may lead to reduced compliance with protocols. Adhering to protocols may conflict with other demands on staff time or be at odds with patients’ other interests. For example, routine night‐time vital sign monitoring interferes with sleep and sleep disruptions are associated with several adverse clinical outcomes (Sharda, Carter, Wingard, & Mehta, 2001; Yoder, Yuen, Churpek, Arora, & Edelson, 2013).

Ward culture and shift patterns seem to play a role in the failure of staff to follow expected protocols for managing deteriorating patients (Shearer et al., 2012). For instance, a prospective study from Scotland regarding the overnight use of a standardized Early Warning Score system in patients who were already cause for clinical concern showed that observations in a combined assessment unit were missing in 38% of patients compared with 64% in a ward covered by the Hospital at Night team. The most common signs omitted were respiratory rate, temperature and neurological status and the calculation of the total warning score also included errors (Gordon & Beckett, 2011). A Belgian study of the introduction of a standardized nurse observation protocol, which included an EWS, found that the patient observation frequency per nursing shift was significantly lower during night shifts (De Meester et al., 2013). Even when an electronic physiological surveillance system (EPSS) was in place to guide the timing of observations in an acute hospital in England (Schmidt et al., 2015; Smith et al., 2006), variations were found in the level of documented observations throughout the whole 24 hours, with fewer observations being performed at night (Hands et al., 2013). The authors noted that at night, “even observations that indicated that the patient was unwell, these were not necessarily followed by a subsequent vital signs assessment at a timely interval” (p. 4). Additionally, a qualitative study in Australia reported a tendency for nurses not to wake patients at night to do full checks of patient status (Endacott, Kidd, Chaboyer, & Edington, 2007).

Given our poor understanding of factors that affect compliance with vital signs protocols in acute hospital settings (Smith, Recio‐Saucedo, & Griffiths, 2017), we developed and conducted a web‐based survey of bedside staff about their views of taking vital signs observations at night. The aims of this study were as follows:

1. to improve the understanding of factors affecting patient surveillance at night through a description of the characteristics of bedside staff (i.e., nurses and midwives) working night shifts and their views in relation to performing vital signs observations and to explore the relationships between healthcare staff characteristics and the conduct of vital signs observations at night.

## 3 DESIGN

This was an exploratory descriptive study using an electronically administered survey of nurses, midwives, student nurses and healthcare assistants in a 1200‐bed NHS acute general hospital Trust in the south of England. The survey was designed at the participating hospital by the Deteriorating Patient Group, a taskforce created to improve hospital practices of patient monitoring.

## 4 METHOD

### 4.1 Survey development

The idea for the survey started in the Deteriorating Patient Group at the hospital trust where the study took place, in response to concerns about audit evidence of widespread poor practice during night‐time observations. The group is mainly composed of senior nurse managers and educators responsible for improving practice on their wards. The impetus for the survey was the lack of agreement on the key factors believed to influence compliance with the observation protocol. Key issues related to in‐hospital patient monitoring during the night, which had been identified through a scoping literature review (Griffiths et al., 2014), were used in the development of a web‐based survey discussed and tested with the group. Testing allowed us to clarify the survey items, confirm accurate interpretation of the survey questions and time the completion of the survey. No data were collected from this exercise as the purpose was to clarify question interpretation, assess the range of responses and confirm completeness of the topics covered in relation to the aims of the study.

A total of 35 questions exploring factors related to nurses’ and midwives’ views of the hospital's EWS protocol and the EPSS implemented across the hospital (VitalPAC^TM^, The Learning Clinic, London) were added to a web‐based survey system (a local installation of SurveyMonkey) provided by the participating hospital. Questions were aimed at capturing the views of staff providing bedside care. Survey responses were collected on a Likert‐type scale with five options: ‐strongly agree‐; ‐agree‐; ‐neither agree nor disagree‐; ‐disagree‐; ‐strongly disagree‐. The survey sought to identify staff (e.g., grade, role, experience) and environmental factors (e.g., ward culture, workload, resources) that might be associated with timely measurement of vital signs.

### 4.2 Data collection

In June 2015, an email invitation to respond to the web‐based survey was sent to approximately 2900 bedside staff registered in the Employee Service Record (ESR) system in the hospital. In addition, it was recognized that student nurses and some agency staff would not have email addresses issued by the hospital Trust. These staff were identified by a representative of the Deteriorating Patient Group and invited to complete the online survey. Two reminders were sent within a month. The survey received 695 responses and 497 (72%) participants met the inclusion criterion of working at least one night shift in the past year. A total of 198 (28%) responders were thanked for responding but instructed not to complete all the survey because their shift patterns did not meet the study inclusion criteria.

## 5 ANALYSIS

The first stage of analysis focused on respondent characteristics, summaries of response frequencies and response consistency. Exploratory factor analysis was used to examine the relationships between variables and the underlying structure of the survey; we identified similarities between items and performed dimensionality reduction to avoid redundancies. In addition to item reduction and correlation, factor analysis offered a way to interpret the data structure. Because the number of dimensions used to represent correlations in the data is a crucial issue in any factor analysis, we followed two techniques to resolve the issue: (a) we extracted principal components until the eigenvalue was less than 1 and (b) we used the very simple structure criterion (Revelle & Rocklin, 1979). In the second stage of analysis, multivariate techniques were used to identify relationships between survey items and to investigate overall internal consistency/reliability on all items (acceptable internal consistency reliability at a value of ≥0.70). Using multivariable regression analysis, we related the estimated factor scores to the reported characteristics of healthcare staff: role, number of night shifts worked and experience; this process enabled us to better characterize the work performed at night and to predict staff characteristics associated with surveillance. Our overall analytical approach extended the method reported by Mok et al. (2015) by using the estimated factor scores as response variables in the regression analysis. Statistical analysis was performed using the computing environment R (version 3.4.0 R Development Core Team, 2017).

### 5.1 Construct validity and reliability

Cronbach's alpha was 0.94 for the 35 items. The overall intraclass correlation coefficient (ICC) was 0.75 (95% CI = 0.721–0.783, *p* < 0.001). ICC results with values higher than 0.90 indicated that some items were redundant and that factor analysis was required to reduce data complexity. To increase confidence that a factor analysis could be performed, we tested for sphericity and sampling adequacy. Bartlett's test of sphericity was statistically significant (χ^2^_595_ = 17104.78, *p*‐value < 0.001) and the overall Kaiser–Meyer–Olkin measure of sampling adequacy was 0.83, confirming that the sample was large enough to perform factor analysis. Here, we report those results of the factor analysis that account for most of the variation.

### 5.2 Multivariable regression analysis

Healthcare staff characteristics were included in the linear predictor. Because the data structure is clearly hierarchical (staff are clustered into wards), scores of staff belonging to the same ward share the same ward‐specific (random) effects and are expected to be more similar than scores randomly selected from different wards. One natural way of representing this clustered/hierarchical structure in the data is by adding an unobserved random effect to the linear predictor. Thus, the clustering dependence is modelled on the same scale as the linear predictor, a natural choice if the dependence is considered to arise from unmodelled heterogeneity due to the omission of one or more important explanatory variables. Here, we considered a linear mixed model where random effects varied across wards but—for a given ward—remained constant for all staff. Furthermore, we adopted a nonparametric maximum likelihood approach (Aitkin, 1999; Böhning, 1995) that allowed us to identify clusters of *similar* wards as a by‐product of the estimation procedure. Formally, for each identified factor, let y_iw_ be the score for staff *i* (i = 1,…,I_w_) in ward *w* (w = 1,…,W) and x_iw_ the set of considered covariates. We defined the following linear mixed regression model:

$$y_{\mathrm{iw}}=x_{\mathrm{iw}}^{\prime }\beta +b_{w}+\varepsilon_{\mathrm{iw}}$$

where β was a vector of fixed regression coefficients, ε_iw_ was the error term and b_w_ was a discrete ward‐specific random effect that accounted for the clustered structure of the data and takes values in a finite set of, say, K values (b_1_,b_2_,…,b_K_). The random effect can be seen as a random ward‐specific intercept. The resulting likelihood function is given by

$$\prod_{w=1}^{W}\sum_{k=1}^{K}\pi_{k}\prod_{i=1}^{I_{w}}f\left(y_{\mathrm{iw}}|x_{\mathrm{iw}},b_{k}\right)$$

which is a finite mixture of distributions with K clusters (or components), each with probability π_k_; where f() is a generic probability density function. Accordingly, wards were clustered into clusters on the basis of the estimated probabilities of having a cluster‐specific intercept. We then treated the ward‐specific intercept and corresponding probabilities as unknown parameters. Parameters estimates were obtained via the maximum likelihood approach by using the Expectation–Maximization algorithm. The number K of clusters is also unknown but is treated as fixed and is sequentially increased until the likelihood is maximized; model selection is performed by using widely known penalized likelihood criteria.

## 6 ETHICS

The study received ethics approval (No 10813) from the University of Southampton committee on 30/03/2015 and governance approval from the Research & Development Office at the participating hospital on 15/06/2015.

## 7 RESULTS

### 7.1 Survey responses and respondent characteristics

Out of the 695 surveys received, 198 responses did not meet the inclusion criteria (working at least one night shift) and were excluded. Of the 497 eligible responses (72%), 24 respondents left 5 items unanswered, 188 left 12 items and 26 responded only to the section on personal characteristics. This resulted in 259 complete records included in the sample for analysis (52% of eligible responses). A sensitivity analysis was performed by imputing the items for the 24 respondents who left five items unanswered. The results did not significantly vary in terms of the interpretation of the factors or factor scores. The characteristics of the valid responses are presented in Table 1. Most were Staff Nurses (SN) (54.1%) and three quarters of the respondents had worked more than 10 night shifts in the last 12 months. Only 16% worked only night shifts. Most (67%) had more than 5 years of work experience.

**Table 1:** Survey respondents

Variable	*N* (%) (Total *N *=* *497)
Role	
Staff nurse	269 (54.1)
Healthcare support worker	120 (24.1)
Senior nurse/manager	52 (10.5)
Student nurse	32 (6.5)
Midwife	24 (4.8)
Number of night shifts worked in the last 12 months	
1–5	75 (15.1)
6–10	49 (9.9)
>10	373 (75.1)
Night duty arrangements	
Night shifts only	81 (16.3)
Occasional night shifts	67 (13.5)
Rotation, including nights	349 (70.2)
Usual number of wards worked on during night shifts	
Only one ward	241 (48.5)
More than one ward	256 (51.5)
Experience (years)	
0–5	152 (30.6)
6–10	94 (18.9)
11–15	71 (14.2)
16–20	62 (12.5)
>20	118 (23.7)

### 7.2 Description of knowledge, beliefs and attitudes of nursing staff influencing observations at night

**Figure 1:**
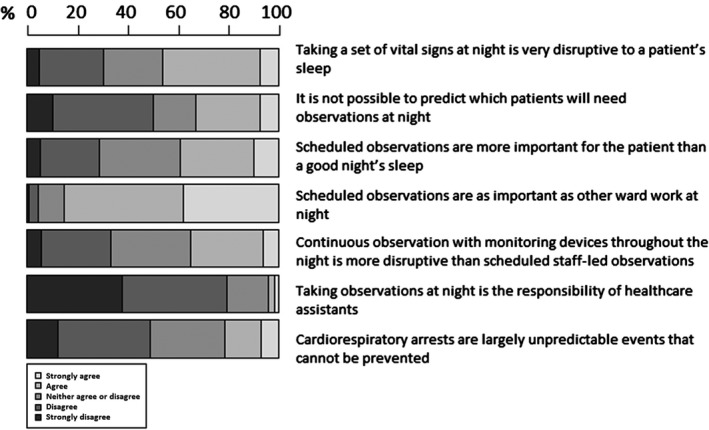
Beliefs influencing monitoring at night

The responses to attitudes and behaviours regarding monitoring at night are displayed in Figure 1. Although 46% of staff agreed that taking vital signs observations at night is very disruptive to sleep, the response that observations were more important than a good night's sleep had slightly more agreement than disagreement (29.4% vs. 23.4%), with many staff neither agreeing nor disagreeing (32.1%). There was a high level of agreement (85.1%) with the statement that scheduled observations were as important as other ward work at night. A proportion of staff agreed that it is difficult to predict which patients would require observations at night and that cardiorespiratory events are unpredictable and cannot be prevented (33% and 21.5%, respectively). The response to whether continuous electronic monitoring was thought to be more disturbing at night than staff observations was almost equivocal. The majority of respondents (79.4%) disagreed that healthcare assistants are responsible for monitoring at night.

**Figure 2:**
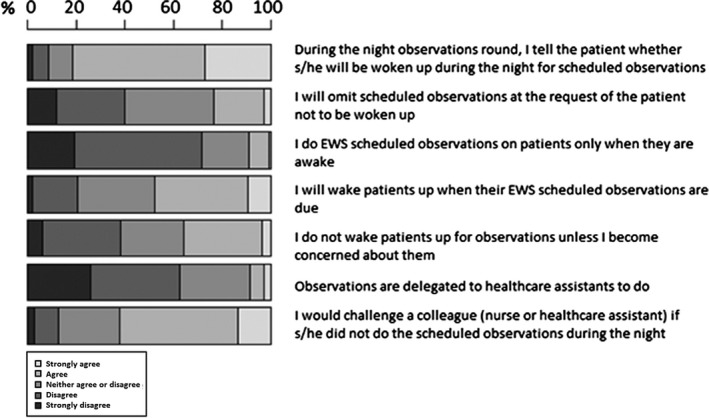
Behaviours influencing compliance with EWS‐scheduled observations at night

Responses relating to behaviours when conducting observations are displayed in Figure 2. The majority (81.6%) of staff would inform patients in advance that they would be awakened at night to take observations and 71.6% disagreed with the statement that they would do observations only if the patient was awake. Similarly, 40% disagreed with the statement that they would omit observations at the patient's request; however, 36.8% neither agree nor disagree and 47.9% agreed, that they would wake up the patient when the EWS was due. The response to waking up the patient only if they were worried was almost the same (38.3% disagreed, 33.8% agreed). In regard to relationships among and behaviour of colleagues, 62.4% disagreed that observations should be delegated to HCSWs and 62.1% agreed that they would challenge colleagues if they did not do the required observations.

**Figure 3:**
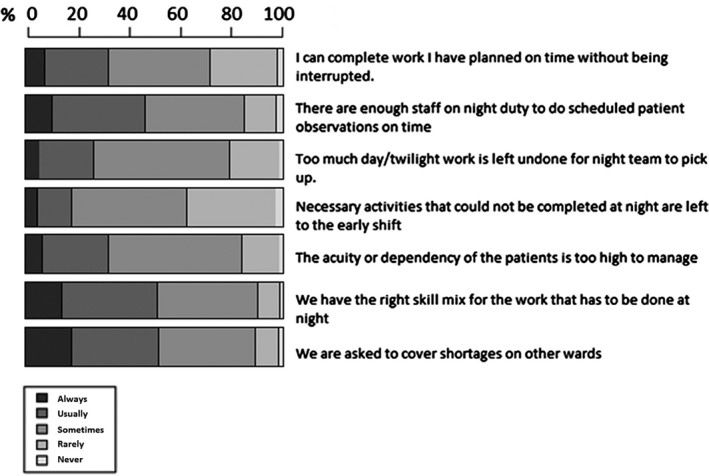
Beliefs about work environment (staffing) and workload affecting observations

Responses regarding beliefs about factors in the work environment (staffing and workload) affecting observations are displayed in Figure 3. Only 32% of staff felt they could always or usually complete planned work on time without being interrupted, but a higher proportion (46.4%) felt there were enough staff on night duty to do scheduled patient observations on time. Regarding work completion between shifts, only 26.4% of staff felt there was always or usually too much day/twilight work left undone for the night team to pick up and 18.3% said they always or usually left necessary activities that could not be completed at night to the day shift. Covering shortages on other wards was common and was reported by 51.3% of respondents as something done always or usually. Only 16.4% of respondents felt patient acuity or dependency was always or usually too high to manage, but 47.9% felt the skill mix was only sometimes or rarely right for the work they were supposed to do at night.

**Figure 4:**
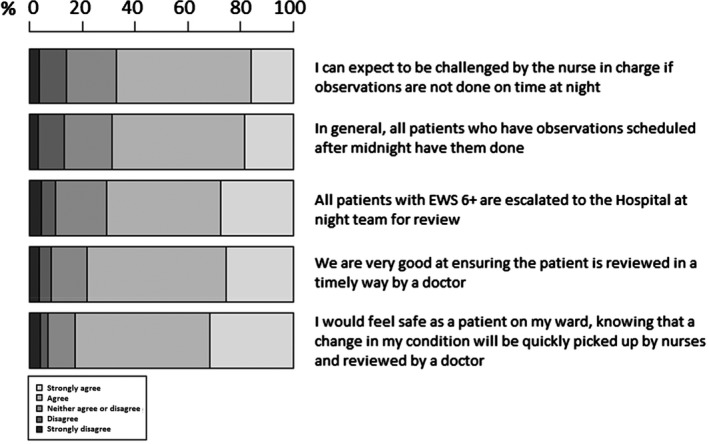
Beliefs about ward efficiency at night with regard to conducting observations

Beliefs about ward efficiency at night with regard to conducting observations are displayed in Figure 4. The majority of respondents (67.1%) agreed they could expect to be challenged by the nurse in charge if observations were not done on time at night and only 13.2% disagreed with the statement that in general, all patients who have observations scheduled after midnight have them done. There was a high level of agreement with statements concerning escalation, senior review and patient safety, with 70.9% agreeing that all patients with EWS ≥6 are escalated to the Hospital at Night team for review, 78.3% agreeing that “we are very good at ensuring the patient is reviewed in a timely way by a doctor” and 83% agreeing that they would feel “safe as a patient on my ward”, knowing that a change in their condition would be quickly picked up by nurses and reviewed by a doctor.

### 7.3 Dimensionality reduction and factor identification

While analysing the data we explored solutions with varying number of latent factors, taking into consideration that too many factors may not summarize the data accurately and yield poor interpretations and too few may not allow the model to represent the true underlying data structure. Our model selection was based on the trade‐off between variance explanation and factors’ interpretation. In choosing the best model, we referred to the scree‐plot and the interpretation of the factors, finding that the five‐factor model increased the explained variance by less than 5% and decreased factor interpretation. Our chosen four‐factor solution explained 60% of the overall variance without compromising interpretation. A summary of the standardized loading of the factors is presented in Table 2.

**Table 2:** Results of factor analysis

	Standardized loadings			
	Factor 1	Factor 2	Factor 3	Factor 4
SS loadings	13.03	3.21	2.63	1.93
Proportion Var	0.37	0.09	0.08	0.06
Cumulative Var	0.37	0.46	0.54	0.60
Proportion explained	0.63	0.15	0.13	0.09
Cumulative Proportion	0.63	0.78	0.91	1.00

	Factor 1: Workload, resources & capacity	Factor 2: Prioritization	Factor 3: Safety culture	Factor 4: Responsibility & control	Commonality	Uniqueness	Hoffman's index of complexity
Factor 1 Workload, resources and capacity							
Overall experience of night shifts worked							
I can complete work on time without interruptions	0.92	−0.06	−0.04	−0.09	0.86	0.14	1
There are enough staff to do scheduled observations on time	0.90	−0.13	−0.16	−0.01	0.85	0.15	1.1
Too much day work is left undone for night staff to pick up	0.94	0.05	−0.04	−0.04	0.88	0.12	1
Necessary activities that could not be completed at night are left to the early shift	0.94	−0.05	−0.02	−0.03	0.88	0.12	1
The acuity or dependency of the patients is too high to manage	0.92	0.02	−0.04	−0.04	0.86	0.14	1
We have the right skill mix for the work that has to be done at night	0.88	−0.09	−0.15	0	0.80	0.2	1.1
We are asked to cover shortages on other wards	0.87	0.07	0.05	−0.01	0.76	0.24	1
On the use of agency staff at night							
Affects how well we can work as a team	0.91	−0.01	−0.05	−0.05	0.84	0.16	1
Agency staff know and follow the EWS protocol	0.93	−0.14	−0.08	0.05	0.89	0.11	1.1
Agency staff have access and know how to use VitalPAC	0.90	−0.11	−0.05	0.07	0.83	0.17	1.1
Affects skill mix on the ward to the extent it affects patient care	0.91	−0.09	−0.04	−0.03	0.83	0.17	1
Affects doing observations on time	0.93	0.03	−0.09	−0.03	0.87	0.13	1
Using VitalPAC on our ward							
At the start of my night shift I can virtually always find an iPod that is sufficiently charged and in working condition	0.85	0.22	0.13	−0.05	0.79	0.21	1.2
iPods connects reliably and fast to the network at night	0.83	0.18	0.13	0.07	0.75	0.25	1.2
More visible display of scheduled observations (for example on a large screen on the ward) would make a difference in compliance with Trust protocol at night	0.89	0.13	0.08	0.07	0.82	0.18	1.1
Overall, the VitalPAC system helps rather than hinders the timely observation of patients	0.90	0.13	0.14	0.01	0.85	0.15	1.1
Factor 2 Prioritization							
Representation of nurses’ attitudes regarding given statements							
Scheduled observations are more important for the patient than a good night's sleep	0.12	0.6	0.02	0.16	0.40	0.6	1.2
Scheduled observations are as important as other ward work at night	0.04	0.48	0.02	−0.13	0.25	0.75	1.2
I will wake patients up when their EWS‐scheduled observations are due	−0.03	0.65	0.16	0.3	0.53	0.47	1.5
I would challenge a colleague (nurse or healthcare assistant) if s/he did not do the scheduled observations during the night	−0.05	0.69	0.03	−0.02	0.49	0.51	1
a Taking a set of vital signs at night is very disruptive to a patient's sleep	−0.01	−0.33	−0.03	0.26	0.18	0.82	1.9
a I often omit scheduled observations at the request of the patient not to be woken up	0.01	−0.59	0.12	0.19	0.40	0.60	1.3
a I do EWS‐scheduled observations on patients only when they are awake	−0.03	−0.63	−0.04	0.16	0.42	0.58	1.1
a I do not wake patients up for observations unless I become concerned about them	0	−0.62	−0.11	0.01	0.40	0.6	1.1
Factor 3 Safety culture							
Views of what happens on the ward with respect to night observations and patient deterioration							
I can expect to be challenged by the nurse in charge if observations are not done on time at night	−0.04	0.25	0.59	−0.12	0.43	0.57	1.5
In general, all patients who have observations scheduled after midnight have them done.	0.01	0.38	0.65	0.14	0.59	0.41	1.7
All patients with EWS 6 + are escalated to the Hospital at Night team for review	−0.03	−0.08	0.55	0.13	0.32	0.68	1.2
We are very good at ensuring the patient is reviewed in a timely way by a doctor	0	0.01	0.8	−0.11	0.65	0.35	1
I would feel safe as a patient on my ward, knowing that a change in my condition will be quickly picked up by nurses and reviewed by a doctor	0.01	0.01	0.82	−0.12	0.68	0.32	1
During the night observations round, I tell the patient whether s/he will be woken up during the night for scheduled observations	−0.05	0.2	0.15	−0.07	0.07	0.93	2.2
Factor 4 Responsibility and control							
Representation of nurses’ attitudes regarding given statements							
It is not possible to predict which patients will need observations at night	0.03	−0.03	−0.09	0.58	0.34	0.66	1.1
Continuous observation with monitoring devices throughout the night is more disruptive than scheduled staff‐led observations	−0.01	−0.07	0.04	0.33	0.12	0.88	1.1
Taking observations at night is the responsibility of healthcare assistants	0.03	0.08	−0.13	0.63	0.42	0.58	1.1
Cardiorespiratory arrests are largely unpredictable events that cannot be prevented	−0.01	−0.07	0.08	0.67	0.46	0.54	1.1
The completion of observations is delegated to healthcare assistants	−0.08	−0.09	−0.03	0.51	0.28	0.72	1.1

a Negative correlation with the factor

Hoffman's index provided information on how many factors are used to explain each variable in the four‐factor solution, which characterizes the core of the questionnaire. Most of the items analysed were explained by one factor only, with very few exceptions. This facilitated an even interpretation of the factors.

### 7.4 Interpretation of factors

Factor 1 Workload, resources and capacity. Factor 1 clustered 16 items that evaluated the adequacy of the work environment related to staffing, skill mix and availability of the EPSS. Items loading ranged between 0.8 and 0.9 and accounted for the 37% of the variance. The clustered items explored staff views on the adequacy of the work environment related to staffing, skill mix and availability of the EPSS (VitalPAC). Survey items asked staff to indicate agreement with statements such as “Too much day/twilight work is left undone for night team to pick up”; “Necessary activities that could not be completed at night are left to the early shift”; “The use of agency staff at night affects the completion of observations on time.”

Factor 2 Prioritization, grouped eight items that explored the characteristics of nurses and wards and the ways these were associated with task priorities and decision‐making about waking patients up to perform observations. Four items were positively associated with this factor, with loadings 0.5‐0.7 and four other items were negatively correlated, with loadings ranging from ‐0.6 to ‐0.3. Its contribution explains 9% of the variability in the data. The results indicated varying decisions among the different groups of nursing staff regarding waking up or allowing patients to have long periods of uninterrupted sleep by delaying due observations.

Factor 3 Safety culture, grouped six items on nurses’ views regarding work to improve patient safety during the night shift. Factor 4, Responsibility and control, incorporated five items that provided an overall description of staff views regarding who is responsible for taking observations and the extent to which nurses felt that adverse events could be predicted during the night. Factors 3 and 4 had loadings of 0.5 and 0.6, which explained variability of 5% and 6%, respectively. Responses covered items including “I expect to be challenged by the nurse in charge if observations are not done on time” or “All patients with EWS 6+ are escalated to the Hospital at Night team for review”. These responses illustrate that nurses are aware of the ward practices and the importance given to completing observations on that ward; both of these items are relevant to the maintenance of a safe environment.

### 7.5 Relationship between factors and healthcare staff characteristics

To understand how responses varied according to nurses’ characteristics, we undertook regression models with the factor scores as response variables and nurses’ characteristics as covariates. The results are presented in Table 3. For all the models, one for each factor, a two‐cluster mixture model is identified. Clusters are formed by homogeneous wards (in some sense) and each ward is associated to cluster k (k = 1,2) with probability π_k_ (k = 1,2). Clusters differ by the cluster‐specific intercepts b_k_, which can be interpreted as different propensities towards the response variable.

**Table 3:** Multiple regression coefficients of healthcare staff characteristics associated with factors, with standard errors in brackets

Covariates	Factor scores (response variables)			
	Workload, resources & capacity	Prioritization	Safety culture	Responsibility & control
Role				
Staff nurse^a^				
Healthcare support workers	0.024 (0.026)	−0.005 (0.105)	−0.164 (0.087)	1.023 (0.097)
Midwives	0.035 (0.063)	0.440 (0.221)	−0.247 (0.257)	0.112 (0.232)
Senior nurse/Manager	−0.060 (0.041)	0.119 (0.164)	−0.022 (0.167)	−0.492 (0.152)
Student nurse	−0.080 (0.040)	0.505 (0.202)	0.242 (0.207)	0.561 (0.187)
No. night shifts worked				
1–5	0.054 (0.033)	0.534 (0.133)	−0.260 (0.125)	0.305 (0.122)
6–10	0.043 (0.036)	0.200 (0.142)	0.137 (0.145)	0.032 (0.131)
>10^a^				
Night duty arrangements				
Rotation, including nights^a^				
Night shifts only	−0.075 (0.030)	−0.116 (0.120)	0.302 (0.123)	0.087 (0.111)
Occasional night shifts	−0.018 (0.037)	−0.291 (0.146)	0.031 (0.152)	−0.390 (0.136)
No. wards during night shifts				
More than one ward	−0.036 (0.021)	0.133 (0.085)	−0.002 (0.087)	0.260 (0.078)
Only one ward^a^				
Experience				
0–5	−0.051 (0.032)	0.165 (0.126)	0.230 (0.109)	0.056 (0.116)
6–10^a^				
11–15	−0.050 (0.037)	0.136 (0.149)	−0.086 (0.152)	0.198 (0.137)
16–20	0.064 (0.029)	0.444 (0.158)	0.175 (0.162)	0.549 (0.145)
>20	0.003 (0.035)	0.262 (0.140)	0.131 (0.144)	−0.001 (0.129)
Intercept group 1 (b_1_)	0.893 (0.029)	−0.606 (0.130)	−0.603 (0.160)	−0.439 (0.108)
Intercept group 2 (b_2_)	1.173 (0.042)	0.058 (0.119)	−0.003 (0.120)	0.021 (0.124)
Group 1 proportion (π_1_)	0.883	0.453	0.144	0.581
Group 2 proportion (π_2_)	0.117	0.547	0.856	0.419

a Reference category: variables found to be most frequent in practice were chosen as comparators.

#### 7.5.1 Factor 1. Workload, resources and capacity

By using the Bayesian information criterion to perform model selection, i.e., to choose the number of clusters, we identified two clusters of wards concerning staff perceptions of having adequate resources and capacity to complete their required workload. All wards grouped into one cluster, with the exception of paediatric wards (π_2_ = 0.117), which shows nurses’ perceptions that resources and capacity on this ward were adequate to complete work on time without interruptions and to complete scheduled observations on time. In comparison with student nurses, more experienced nurses (i.e., 16–20 years of work experience) had higher scores. Higher scores from staff working on a rotational basis indicated a greater likelihood to consider resources and capacity as a crucial aspect of work. Other staff characteristics were not significant.

#### 7.5.2 Factor 2. Prioritization

The results of the analysis of the prioritization factor converged in two clusters of wards: geriatric wards (including rehabilitation) and surgical and respiratory wards. The two clusters were clearly different, as shown by the two cluster‐specific intercepts (b_1_ = −0.027, b_2_ = 0.637), where wards in the second cluster were more likely to follow the EWS protocol and those in the first cluster were slightly more inclined to follow individual knowledge. With regard to nurses’ characteristics and the priority of waking patients to take observations, student nurses’ and midwives’ responses showed an inclination to follow the protocol more than HCSWs; while those working more often at night and those working only occasionally at night had the perception that individual knowledge was more important than the protocol.

#### 7.5.3 Factor 3. Safety culture

Strategies to maintain a safety culture scored higher for nurses working more often at night and for student nurses. Nurses who work at night only occasionally or on a rotational basis, as well as HCSWs, scored lower on the items that captured how much the staff agreed with using only the EWS protocol to ensure patient safety. Wards were similar with regard to this factor, as only a few (e.g., acute medical unit and general surgery) had lower propensities (i.e., intercepts) on items about including patient escalation and following the EWS protocol.

#### 7.5.4 Factor 4. Responsibility and control

Major differences were observed for items correlating responsibility and control scores with the following nurses’ characteristics: role, experience, number of wards worked in and in the frequency of night shifts. Nurse's role, the number of night shifts worked and night duty arrangements were associated with the perception of staff being responsible for taking observations (e.g., taking observations at night is the responsibility of healthcare assistants) and control over predicting adverse events (e.g., the belief that cardiorespiratory arrests are largely unpredictable events that cannot be prevented). Having control of tasks at night was ward‐specific. Variability did not allow for clustering wards per specialty or function. The way items regarding responsibility and control were ward‐specific were thus influenced by staff characteristics.

## 8 DISCUSSION

This study aimed to improve our understanding of patient surveillance during the night shift at an acute hospital and to identify the associations between staff characteristics and timely completion of vital signs observations following an EWS protocol. We designed a survey to collect staff knowledge, beliefs and attitudes concerning the night shift and the use of the EWS to complete scheduled observations. Factor analysis was used to assess and validate the theoretical construct, internal reliability and accuracy of the survey to measure factors that influence staff completion of vital signs observations during the night shift.

Descriptive analysis of the online survey revealed that 54% of staff disagreed with the statement that taking a set of vital signs observations at night is very disruptive to sleep, which is consistent with most staff agreeing that they would wake patients up to conduct observations. However, only half of the respondents agreed to wake up patients when EWS‐scheduled observations were due. This behaviour may hint at staff relying on clinical judgement to follow EWS‐scheduled observations during the night. Only 33% of staff agreed with the statement that it is difficult to predict which patients would require observations and 21.5% agreed with the statement that cardiorespiratory arrests are largely unpredictable events that cannot be prevented, suggesting that the role of the EWS as shorthand for the individual patient's risk of physiological deterioration leading to avoidable cardiac arrest may not be fully appreciated by all staff (Kause et al., 2004).

When interpreting these findings, the roles played by ward factors that affect the availability of staff to complete care tasks must be considered. These factors include organization of nursing care activities, staffing levels, skill mix and workload (Hogan, 2006; James, Butler‐Williams, Hunt, & Cox, 2010). The results also showed that workload associated with overall patient acuity did not seem to be a factor affecting observations and 46.4% felt there were enough staff on the night shift to complete scheduled patient observations on time. However, 47.9% felt the skill mix was rarely or never appropriate for the work that had to be completed during the night shift. This response was contested by similar proportions of staff who responded “always” or “usually” to similar statements related to staffing. Though it might be counterintuitive at first glance, this reflects the multidimensionality of care: what is sufficient to guarantee a reasonable quality of work in general might not be when specific tasks are required, leading to prioritizing tasks and ultimately neglecting care (Ball, Murrells, Rafferty, Morrow, & Griffiths, 2014). These seemingly mixed beliefs from the respondents require further exploration to fully understand the effect of staffing on monitoring patients at night.

There was a high level of agreement with statements concerning escalation, senior review and patient safety. The majority of respondents agree that HCSWs are not responsible for conducting observations (79.4%); however, fewer respondents indicated that observations should not be delegated to HCSWs (62.4%), which resonates with findings from studies exploring nursing staff practices of conducting observations (Wheatley, 2006). We argue that the conflict in these responses may be due to ward characteristics related to what decisions are made when workload on the wards is too high to manage, the skills of individual HCSWs, or even the lack of clarity regarding whose responsibility it is to conduct observations (Thornley, 2000).

The results of the factor analysis grouped survey responses into 4 factors: workload and resources, prioritization, safety culture and responsibility and control. Responses to these factors were associated with staff or ward characteristics. Regression results indicated that staff role, experience and number of night shifts worked were associated with completion of care tasks. Nurses with more years of experience (16 and 20 years) believed workload and capacity were factors that had an impact on the completion of observations at night. However, results for nurses with more than 20 years of experience were not significant. All categories of years of experience were significant for the prioritization factor, indicating that more experienced staff based their decisions to conduct observations more on knowledge (clinical judgement) (Mann, 2012) and less on the EWS system.

Associations of shift patterns with the prioritization factor were not significant, showing that the number of night shifts worked did not play a role in decisions about completing observations. In regard to staff role, student nurses and midwives were associated with prioritization of observations at night, indicating that ward culture or practices, in addition to experience, inform decisions about conducting observations during the night shift. This was also evident in the way that paediatric wards were the exception in the associations of characteristics with the workload and resources factors. Regression results showed that paediatric wards differed from the other wards with regard to adequate resources, implying that staff perceived that they had enough resources to complete observations. With regard to safety culture, questions about completing observations for high acuity patients, escalation to the Hospital at Night team or feeling safe on the ward because of prompt monitoring and action, were significant for student nurses, staff working mostly night shifts and staff with either low (<5) or high numbers of years of work experience (>30).

The findings reported here were examined in a qualitative study aimed at exploring factors related to adherence with the EWS protocol at night. The study by Hope et al., 2018 complemented the picture presented here, showing that the EWS focus on preventing deterioration required fine‐tuning to incorporate exceptions created by patients requiring long‐term management or those in palliative care trajectories (Hope et al., 2018). The approach of calculating EWS without considering variations in patient groups with conditions such as COPD or dementia resulted in observations being determined more by clinical judgement than by the EWS.

## 9 LIMITATIONS

This study was conducted in a single hospital; therefore, the results may not necessarily be transferable to other organizations. There is also a potential bias from the way the questions were worded and the design and structure of the survey. The risk of responder bias (i.e., reluctance or inability to respond honestly and accurately) was minimized by exploring the same question in separate items. A limitation of the analysis was that the sample was created with complete surveys only, which could also introduce bias. However, the majority of the excluded records were left almost entirely unanswered. Multiple imputation would have led to an increase in uncertainty without adding any significant information to the analysis. To strengthen the rigour of the survey for further research, we recommend undertaking convergent and discriminant validity to examine the similarities and differences of the questionnaire with other tools. It is also recommended that structural equation modelling and confirmatory factor analysis be undertaken in a larger sample to support the generalizability of the results (Watson & Thompson, 2006).

## 10 CONCLUSION

The widespread implementation of EWS protocols, along with its reported impact on patient outcomes thanks to early identification of deterioration (Alam et al., 2014; McNeill & Bryden, 2013), highlights the relevance of this study to health organizations in contexts outside the UK, especially when evidence suggests that at night, patients are not being monitored with the frequency indicated by the protocol. The results of this study revealed the multiple factors involved in the decisions made by staff to complete observations at night‐time following the EWS. The fact that staff and ward characteristics play a role in the decision to complete observations raises the need to review the surveillance protocols in medical and surgical wards where foregoing observations may be compromising patient safety. In addition, ward heterogeneity may influence nurses’ surveillance attitudes and behaviours. Missed observations may be due not only to workload and resources but also to nurses’ roles and responsibilities on the ward. Role, experience and shift arrangements can all affect care at night, leading to subjective provision of care and/or to a lack of patient monitoring.

## CONFLICTS OF INTEREST

VitalPAC is a collaborative development of The Learning Clinic Ltd (TLC) and Portsmouth Hospitals NHS Trust (PHT). Drs Schmidt and Meredith are employed by PHT. Professor Smith was an employee of PHT until 31 March 2011. Dr Schmidt and the wife of Professor Smith were TLC shareholders until October 2015. Professor Smith acted as expert advisor to the National Institute for Health and Clinical Excellence during the development of the NICE clinical guideline 50: “Acutely ill patients in hospital: recognition of and response to acute illness in adults in hospital”. He was also a member of the National Patient Safety Agency Committee that wrote the reports: “Recognising and responding appropriately to early signs of deterioration in hospitalized patients” and “Safer care for the acutely ill patient: learning from serious incidents”. No other conflict of interest has been declared by the other authors.

## References

[nop2179-bib-0001] Aitkin, M. (1999). A general maximum likelihood analysis of variance components in generalized linear models. Biometrics, 55(1), 117–128. 10.1111/j.0006-341X.1999.00117.x 11318145

[nop2179-bib-0002] Alam, N. , Hobbelink, E. , van Tienhoven, A. , van de Ven, P. , Jansma, E. , & Nanayakkara, P. (2014). The impact of the use of the Early Warning Score (EWS) on patient outcomes: A systematic review. Resuscitation, 85(5), 587–594. 10.1016/j.resuscitation.2014.01.013 24467882

[nop2179-bib-0003] Ball, J. E. , Murrells, T. , Rafferty, A. M. , Morrow, E. , & Griffiths, P. (2014). ‘Care left undone’ during nursing shifts: Associations with workload and perceived quality of care. BMJ Qual Saf, 23(2), 116–125. 10.1136/bmjqs-2012-001767 PMC3913111 23898215

[nop2179-bib-0004] Böhning, D. (1995). A review of reliable algorithms for the semi‐parametric maximum likelihood estimator of a mixture distribution. Journal of Statistical Planning and Inference, 47, 5–28. 10.1016/0378-3758(94)00119-G

[nop2179-bib-0005] Buist, M. , & Stevens, S. (2013). Patient bedside observations: What could be simpler? BMJ Qual Saf, 22(9), 699–701. 10.1136/bmjqs-2013-002143 23728119

[nop2179-bib-0006] De Meester, K. , Das, T. , Hellemans, K. , Verbrugghe, W. , Jorens, P. G. , Verpooten, G. A. , & van Bogaert, P. (2013). Impact of a standardized nurse observation protocol including MEWS after Intensive Care Unit discharge. Resuscitation, 84(2), 184–188. 10.1016/j.resuscitation.2012.06.017 22796310

[nop2179-bib-0007] Endacott, R. , Kidd, T. , Chaboyer, W. , & Edington, J. (2007). Recognition and communication of patient deterioration in a regional hospital: A multi‐methods study. Australian Critical Care, 20(3), 100–105. 10.1016/j.aucc.2007.05.002 17627836

[nop2179-bib-0008] Gordon, C. F. , & Beckett, D. J. (2011). Significant deficiencies in the overnight use of a Standardised Early Warning Scoring system in a teaching hospital. Scottish Medical Journal, 56(1), 15–18. 10.1258/smj.2010.010009 21515526

[nop2179-bib-0009] Griffiths, P. , Recio Saucedo, A. , Schmidt, P. , & Smith, G. (2014). Vital signs monitoring in hospitals at night. Nursing Times, 111(36–37), 16–17. 26434188

[nop2179-bib-0010] Hands, C. , Reid, E. , Meredith, P. , Smith, G. B. , Prytherch, D. R. , Schmidt, P. E. , & Featherstone, P. I. (2013). Patterns in the recording of vital signs and early warning scores: Compliance with a clinical escalation protocol. BMJ Qual Saf, 22(9), 719–726. 10.1136/bmjqs-2013-001954 23603474

[nop2179-bib-0011] Hogan, J. (2006). Why don't nurses monitor the respiratory rates of patients? British Journal of Nursing, 15(9), 489–492. https://doi.org/10.12968/bjon.2006.15.9.21087 16723921 10.12968/bjon.2006.15.9.21087

[nop2179-bib-0012] Hope, J. , Recio Saucedo, A. , Fogg, C. , Griffiths, P. , Smith, G. , Westwood, G. , & Schmidt, P. (2018). A fundamental conflict of care: Nurses’ accounts of balancing sleep with taking vital signs observations at night. Journal of Clinical Nursing, 27, 1860–1871. 10.1111/jocn.14234 29266489 PMC6001445

[nop2179-bib-0013] James, J. , Butler‐Williams, C. , Hunt, J. , & Cox, H. (2010). Vital signs for vital people: An exploratory study into the role of the Healthcare Assistant in recognising, recording and responding to the acutely ill patient in the general ward setting. Journal of Nursing Management, 18(5), 548–555. 10.1111/j.1365-2834.2010.01086.x 20636503

[nop2179-bib-1000] Kause, J. , Smith, G. , Prytherch, D. , Parr, M. , Flabouris, A. , & Hillman, K. (2004). A comparison of antecedents to cardiac arrests, deaths and emergency intensive care admissions in Australia and New Zealand, and the United Kingdom—the ACADEMIA study. Resuscitation, 62(3), 275–282. 15325446 10.1016/j.resuscitation.2004.05.016

[nop2179-bib-0014] Le Lagadec, M. D. , & Dwyer, T. (2017). Scoping review: The use of early warning systems for the identification of in‐hospital patients at risk of deterioration. Australian Critical Care, 30(4), 211–218. 10.1016/j.aucc.2016.10.003 27863876

[nop2179-bib-0015] Mann, J. (2012). Critical thinking and clinical judgment skill development in baccalaureate nursing students. The Kansas Nurse, 87(1), 26–30.

[nop2179-bib-0016] McNeill, G. , & Bryden, D. (2013). Do either early warning systems or emergency response teams improve hospital patient survival? A systematic review Resuscitation, 84(12), 1652–1667. 10.1016/j.resuscitation.2013.08.006 23962485

[nop2179-bib-0017] Mok, W. , Wang, W. , Cooper, S. , Ang, E. N. K. , & Liaw, S. Y. (2015). Attitudes towards vital signs monitoring in the detection of clinical deterioration: Scale development and survey of ward nurses. International Journal for Quality in Health Care, 27(3), 207–213. 10.1093/intqhc/mzv019 25888564

[nop2179-bib-0018] National Institute for Clinical Excellence . (2007). Acutely ill patients in hospital: recognition of and response to acute illness in adults in hospital NICE, Guidance/Clinical Guidelines CG50. London 21204323

[nop2179-bib-0019] Revelle, W. , & Rocklin, T. (1979). Very simple structure: An alternative procedure for estimating the optimal number of interpretable factors. Multivariate Behavioral Research, 14(4), 403–414. 10.1207/s15327906mbr1404_2 26804437

[nop2179-bib-0020] Schmidt, P. E. , Meredith, P. , Prytherch, D. R. , Watson, D. , Watson, V. , Killen, R. M. , … Smith, G. B. (2015). Impact of introducing an electronic physiological surveillance system on hospital mortality. BMJ Quality & Safety, 24(1), 10–20. 10.1136/bmjqs-2014-003073 25249636

[nop2179-bib-0021] Sharda, S. , Carter, J. , Wingard, J. , & Mehta, P. (2001). Monitoring vital signs in a bone marrow transplant unit: Are they needed in the middle of the night? Bone Marrow Transplantation, 27(11), 1197–1200. 10.1038/sj.bmt.1703052 11551031

[nop2179-bib-0022] Shearer, B. , Marshall, S. , Buist, M. D. , Finnigan, M. , Kitto, S. , Hore, T. , … Ramsay, W. (2012). What stops hospital clinical staff from following protocols? An analysis of the incidence and factors behind the failure of bedside clinical staff to activate the rapid response system in a multi‐campus Australian metropolitan healthcare service. BMJ Quality & Safety, 21, 569–575. bmjqs‐2011‐000692. 10.1136/bmjqs-2011-000692 PMC3382445 22626737

[nop2179-bib-0023] Smith, G. B. (2010). In‐hospital cardiac arrest: Is it time for an in‐hospital ‘chain of prevention’? Resuscitation, 81(9), 1209–1211. 10.1016/j.resuscitation.2010.04.017 20598425

[nop2179-bib-0024] Smith, G. B. , Prytherch, D. R. , Schmidt, P. , Featherstone, P. I. , Knight, D. , Clements, G. , & Mohammed, M. A. (2006). Hospital‐wide physiological surveillance–a new approach to the early identification and management of the sick patient. Resuscitation, 71(1), 19–28. 10.1016/j.resuscitation.2006.03.008 16945465

[nop2179-bib-0025] Smith, G. B. , Recio‐Saucedo, A. , & Griffiths, P. (2017). The measurement frequency and completeness of vital signs in general hospital wards: An evidence free zone? International Journal of Nursing Studies, 74, A1–A4. 10.1016/j.ijnurstu.2017.07.001 28701265

[nop2179-bib-0026] Thornley, C. (2000). A question of competence? Re‐evaluating the roles of the nursing auxiliary and health care assistant in the NHS. Journal of Clinical Nursing, 9(3), 451–458. 10.1046/j.1365-2702.2000.00398.x 11235321

[nop2179-bib-0027] Tysinger, E. L. (2015). How Vital Are Vital Signs? A systematic review of vital sign compliance and accuracy in nursing. Journal of Science & Medicine, 1(1), 68–75.

[nop2179-bib-0028] Watson, A. , Skipper, C. , Steury, R. , Walsh, H. , & Levin, A. (2014). Inpatient nursing care and early warning scores: A workflow mismatch. Journal of Nursing Care Quality, 29(3), 215–222. 10.1097/NCQ.0000000000000058 24569518

[nop2179-bib-0029] Watson, R. , & Thompson, D. R. (2006). Use of factor analysis in Journal of Advanced Nursing: Literature review. Journal of Advanced Nursing, 55(3), 330–341. 10.1111/j.1365-2648.2006.03915.x 16866827

[nop2179-bib-0030] Wheatley, I. (2006). The nursing practice of taking level 1 patient observations. Intensive and Critical Care Nursing, 22(2), 115–121. 10.1016/j.iccn.2005.08.003 16274994

[nop2179-bib-0031] Yeung, M. S. , Lapinsky, S. E. , Granton, J. T. , Doran, D. M. , & Cafazzo, J. A. (2012). Examining nursing vital signs documentation workflow: Barriers and opportunities in general internal medicine units. Journal of Clinical Nursing, 21(7–8), 975–982. 10.1111/j.1365-2702.2011.03937.x 22243491

[nop2179-bib-0032] Yoder, J. C. , Yuen, T. C. , Churpek, M. M. , Arora, V. M. , & Edelson, D. P. (2013). A prospective study of nighttime vital sign monitoring frequency and risk of clinical deterioration. JAMA Internal Medicine, 173(16), 1554–1555. 10.1001/jamainternmed.2013.7791 23817602 PMC3773251

